# Biodegradative potential of fungal isolates from sacral ambient: *In vitro* study as risk assessment implication for the conservation of wall paintings

**DOI:** 10.1371/journal.pone.0190922

**Published:** 2018-01-08

**Authors:** Nikola Unković, Ivica Dimkić, Miloš Stupar, Slaviša Stanković, Jelena Vukojević, Milica Ljaljević Grbić

**Affiliations:** 1 Department for Algology, Mycology and Lichenology, Institute of Botany and Botanical Garden “Jevremovac”, Faculty of Biology, University of Belgrade, Belgrade, Serbia; 2 Department for Microbiology, Institute of Botany and Botanical Garden “Jevremovac”, Faculty of Biology, University of Belgrade, Belgrade, Serbia; Leibniz-Institut fur Naturstoff-Forschung und Infektionsbiologie eV Hans-Knoll-Institut, GERMANY

## Abstract

The principal purpose of the study was to evaluate *in vitro* the potential ability of fungal isolates obtained from the painted layer of frescoes and surrounding air to induce symptoms of fresco deterioration, associated with their growth and metabolism, so that the risk of such deterioration can be precisely assessed and appropriate conservation treatments formulated. Biodegradative properties of the tested microfungi were qualitatively characterized through the use of a set of special agar plates: CaCO_3_ glucose agar (calcite dissolution), casein nutrient agar (casein hydrolysis), Czapek-Dox minimal medium (pigment secretion); and Czapek-Dox minimal broth (acid and alkali production). Most of the tested isolates (71.05%) demonstrated at least one of the degradative properties, with *Penicillium bilaiae* as the most potent, since it tested positive in all four. The remaining isolates (28.95%) showed no deterioration capabilities and were hence considered unlikely to partake in the complex process of fungal deterioration of murals via the tested mechanisms. The obtained results clearly indicate that utilization of fast and simple plate assays can provide insight into the biodegradative potential of deteriogenic fungi and allow for their separation from allochthonous transients, a prerequisite for precise assessment of the amount of risk posed by a thriving mycobiota to mural paintings.

## Introduction

All works of art are susceptible to microbially induced decay. From primitive decorations made out of mollusc shells around 75.000 years BC through paintings in caves of the Upper Palaeolithic and works of Gothic and Renaissance art to contemporary art of the 21^st^ century, the creativity and personal expression of artists have largely involved the application of a wide range of natural materials. For example, *al fresco* wall paintings were prepared with variously coloured mineral powders mixed with lime or pure water and applied to fresh lime plaster on the wall. However, in the case of tempera, paints were made by mixing mineral powders with some natural binders, substances such as oils from seeds (of poppy, flax, and hemp), casein, egg yolk, starch, etc., and applied to already formed dry mortar on the wall [1, 2]. Aside from being suitable substrates for microbial growth, microorganisms pose a serious threat to artworks, as they are ubiquitous in the environment, quiescent, and just waiting for adequate environmental conditions under which to develop. Changes, as minor as a subtle short-term increase in humidity or as major as those caused by floods, can lead to microbial infestations within hours. Among various microorganisms, fungi, now thought to number potentially up to 5.1 million species [3], are considered primary causes of biodeterioration of wall paintings and other works of art [4]. Cosmopolitan, ubiquitous, and capable of colonizing a large number of microhabitats, they are organisms which due to their pronounced enzymatic activity and ability to grow on substrates with low *a*_*w*_ values are able to degrade all the organic and inorganic components artworks are made of, usually resulting in aesthetically unacceptable changes [5]. In numerous studies conducted in the last few decades on mural paintings within historic buildings, churches and hypogea, fungi found to thrive on painted layers or in the interspace between mortar and paint were usually ascomycetes, while zygomycetes and basidiomycetes were less frequent on murals, though abundant in the surrounding environment [6, 7, 8, 9, 10]. However, although the diversity and origin of fungal contaminants of wall paintings are now well known, studies that apart from, research into mycobiota constituents were aimed at obtaining a better understanding of the complex process of fungal-induced deterioration are few and far between, often as a result of the inability to study the process without compromising the structural integrity of wall paintings, but also due to incomplete knowledge of the spectrum of fungi that are able to degrade this unique type of substrate. In order to transcend this issue, data on the diversity of fungi on wall paintings should be complemented with research into the physiological properties of isolated fungi, as insight into the spectrum of physiological features of common colonizers is a prerequisite for precise assessment of the amount of risk they pose to mural paintings [11].

The principal purpose of the present research was to evaluate *in vitro* the deteriorative potential of fungal isolates initially obtained from painted layers of the wall paintings and surrounding air of the old Church of the Holy Ascension in Veliki Krčimir (Serbia), either via cultivation on a set of unique nutrient media that emulate inorganic (limestone) and organic (casein) components of wall paintings or by testing the ability of isolates to secrete metabolites (acidic and alkaline metabolites, organic pigments) with negative effects on the structural and/or aesthetic integrity of mural paintings. The action of “harmful” metabolites and secretion of organic acids that cause considerable acidification of the substrate are among the primary mechanisms through which fungi are involved in the biodeterioration of objects comprising our cultural heritage. The type of produced acid is regulated by available carbon sources and is often dictated by the presence of metal ions in the pigments used [12]. Adverse effects of acids include dissolution of cations and chelation of metal ions from mortar and mineral pigments, which leads to formation of stable metal complexes whose crystallization in the painted layer and mortar causes an increase of internal pressure that results in cracking, peeling, and eventual loss of mural fragments [13]. Furthermore, acidification of the substrate also stimulates fungal growth and accelerates chemical dissimilatory biodeterioration through oxidation, reduction, and transformation of metal ions [7, 14]. This mechanism of mural deterioration is not unique to fungi, but rather occurs with all acid-producing microorganisms; however, since fungi possess a pronounced ability to secrete acids, they are usually considered the main deteriogens of mineral substrata [15]. The selection of casein as a proteinaceous substrate for screening the fungal proteolytic potential was made based on the fact that casein was commonly used as a natural binder for preparation of paint in *al secco* technique [1,5]. Casein applied to canvas cloth was also used to reinforce frescoes [16]. Furthermore, casein even today is used as casein-water in restoration treatments, to repair damage to fresco structures, and it is sometimes added to lime mortar as a consolidant [17]. In Serbia, a consolidant frequently in use for wall painting conservation is called “casein-acrylic binder” or “lime-based binder with additives”, and it consists of 55% lime binder, 27% casein, 14% filler, and 4% concentrated acrylic binder [18]. This is problematical, since casein not only is a source of nutrients for fungal growth, but also acts as an activator of spore germination, which explains the increased bioreceptivity of *al secco* wall paintings, while peptide degradation can lead to pH increase and secondary CaCO_3_ precipitation [19, 20]. Finally there is now a growing interest in fungal-produced pigments as biologically active substances or colourants in the food and textile industries; however, from a conservation standpoint, synthesis and secretion of pigments into the substrate and resulting aesthetic and structural damage to objects of cultural heritage is a burning issue. Pigments from melanin, quinone, mycosporine, hydroxyanthraquinone, and carotenoid groups synthesized to tolerate unfavourable conditions (high UV radiation, thermal stress, and dehydration) and secreted into the substrate interact with its components to bring about undesirable changes in the characteristics and quality of materials of works of art [5, 12, 21]. These changes are usually manifested as alterations in the original colouration of the substrate, with the colour of stains depending not only on that of the secreted pigment, but also on a set of other factors, such as the environmental conditions, chemical composition of the substrate, and interactions between pigments and substrate components [19, 22]. For conservators and restorers, removal of these stains is very difficult, since mechanical cleaning combined with biocidal treatment only removes evident fungal growth, whereas the products of their metabolism, i.e., very stable organic pigments, remain even when the organisms that caused them are completely eliminated [23].

Seeing as how the ability of fungi to degrade organic and inorganic components of wall paintings and produce and secrete various metabolites is clearly troublesome, a knowledge of their potential ability to induce symptoms of deterioration, gained through utilization of fast and simple plate assays will allow recognition of deteriogenic fungi, thereby enabling a precise assessment of risk to be made and appropriate conservation treatments to be formulated and implemented.

## Materials and methods

### Tested fungal isolates

**Fig 1:**
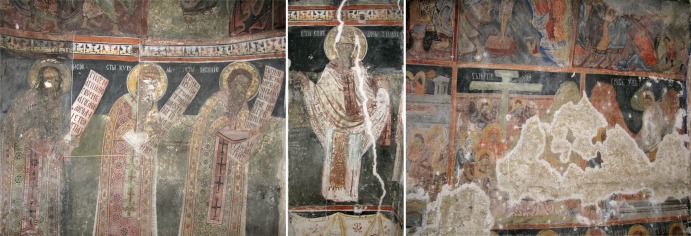
Wall paintings decorating nave walls of the old Church of the Holy Ascension in Veliki Krčimir, Serbia.

All 38 micromycetes screened for their biodegradative potentials are original isolates obtained from deteriorated frescoes (Fig 1) and surrounding air of the nave of the old Church of the Holy Ascension (Veliki Krčimir, Serbia) (Table 1) [24]. Surface isolates were obtained from areas of wall paintings with documented biodeteriorative symptoms (discolouration, exfoliation, cracking, pilling, etc.) and where active fungal growth was confirmed via *in situ* optical and scanning electron microscopy [25, 26]. Likewise, viable fungal propagules dispersed in the air were also sampled and tested, since favourable microclimatic conditions for mould proliferation are present throughout the year [25], resulting in a high spore overload. Fungal isolates were previously determined by ITS and β-tubulin gene sequencing and maintained at −75°C with 1.5 ml of 30% glycerin in cryovials deposited in the Mycotheca of the University of Belgrade—Faculty of Biology (BEOFB).

**Table 1:** Biodegradative potential of fungal isolates from the sacral ambient of the old Church of the Holy Ascension.

No.	Fungal isolates	Code	Origin	Biodegradative assays			
				Pigment secretion	Calcite dissolution	Casein hydrolysis	pH
1	*Alternaria alternata* (Fr.) Keissl.	BEOFB 211m	fresco	-	-	-	6.24 ± 0.13
2	*Alternaria alternata* (Fr.) Keissl.	BEOFB 212m	air	-	-	-	7.08 ± 0.06
3	*Alternaria infectoria* E.G. Simmons	BEOFB 221m	air	-	-	-	7.04 ± 0.02
4	*Aspergillus aureolatus* Munt.-Cvetk. & Bata	BEOFB 361m	fresco	s.YG	-	+	7.03 ± 0.12
5	*Aspergillus creber* Jurjevic, S.W. Peterson & B.W. Horn	BEOFB 371m	fresco	s.O	-	-	7.06 ± 0.03
6	*Aspergillus europaeus* Hubka, A. Nováková, Samson, Houbraken, Frisvad & M. Kolařík	BEOFB 381m	fresco	-	+	+	7.08 ± 0.08
7	*Aspergillus flavipes* (Bainier & R. Sartory) Thom & Church	BEOFB 391m	fresco	l.OY	-	-	7.19 ± 0.06
8	*Aspergillus flavus* Link	BEOFB 313m	fresco	-	-	+	7.09 ± 0.02
9	*Aspergillus niger* Tiegh.	BEOFB 343m	fresco	-	+	+	2.81 ± 0.47
10	*Aspergillus oryzae* (Ahlb.) Cohn	BEOFB 3101m	fresco	-	-	+	7.02 ± 0.11
11	*Aspergillus ostianus* Wehmer	BEOFB 351m	fresco	-	-	+	7.21 ± 0.02
12	*Aspergillus pallidofulvus* Visagie, Varga, Frisvad & Samson	BEOFB 3111m	fresco	-	-	-	6.11 ± 0.12
13	*Aspergillus parasiticus* Speare	BEOFB 3121m	fresco	-	-	-	6.21 ± 0.07
14	*Aspergillus versicolor* (Vuill.) Tirab.	BEOFB 3131m	air	deep rO	-	+	7.04 ± 0.06
15	*Bionectria byssicola* (Berk. & Broome) Schroers & Samuels	BEOFB 401m	air	-	-	-	7.07 ± 0.12
16	*Bjerkandera adusta* (Willd.) P. Karst.	BEOFB 1601	air	-	-	+	7.01 ± 0.03
17	*Chaetomium ancistrocladum* Udagawa & Cain	BEOFB 711m	fresco	m.YG	-	+	7.01 ± 0.02
18	*Chaetomium murorum* Corda	BEOFB 721m	fresco	s.P	-	+	6.25 ± 0.12
19	*Chaetomium murorum* Corda	BEOFB 722m	fresco	s.P	-	-	6.95 ± 0.13
20	*Cladosporium cladosporioides* (Fresen.) G.A. de Vries	BEOFB 1821m	fresco	-	-	+	6.58 ± 0.07
21	*Cladosporium oxysporum* Berk. & M.A. Curtis	BEOFB 1831m	air	-	-	+	6.31 ± 0.21
22	*Cladosporium uredinicola* Speg.	BEOFB 1841m	fresco	m.OlG	-	+	6.56 ± 0.11
23	*Epicoccum nigrum* Link	BEOFB 1701m	fresco	v.rO	-	-	6.98 ± 0.00
24	*Epicoccum nigrum* Link	BEOFB 1702m	air	deep O	-	-	8.31 ± 0.17
25	*Gibberella moniliformis* Wineland	BEOFB 2501m	air	l.OY	-	-	8.47 ± 0.21
26	*Penicillium bilaiae* Chalab.	BEOFB 1131m	air	brill.Y	+	+	2.94 ± 0.33
27	*Penicillium commune* Thom	BEOFB 1141m	air	-	+	-	7.14 ± 0.15
28	*Penicillium griseofulvum* Dierckx	BEOFB 1151m	fresco	-	-	+	5.62 ± 0.23
29	*Penicillium lanosum* Westling	BEOFB 1161m	air	brill.OY	+	-	7.04 ± 0.09
30	*Penicillium lanosum* Westling	BEOFB 1162m	fresco	brill.OY	+	-	7.03 ± 0.02
31	*Penicillium manginii* Duché & R. Heim	BEOFB 1171m	air	v.R	-	-	7.03 ± 0.11
32	*Penicillium rubens* Biourge	BEOFB 1181m	air	-	+	-	6.98 ± 0.09
33	*Phaeosphaeria avenaria* Shoemaker & C.E. Babc.	BEOFB 2001m	air	-	-	+	7.09 ± 0.09
34	*Phoma medicaginis* Malbr. & Roum.	BEOFB 2101m	fresco	-	-	-	7.03 ± 0.12
35	*Phoma medicaginis* Malbr. & Roum.	BEOFB 2102m	fresco	-	-	-	7.05 ± 0.11
36	*Sclerotinia sclerotiorum* (Lib.) de Bary	BEOFB 2201m	air	-	-	-	7.01 ± 0.03
37	*Seimatosporium lichenicola* (Corda) Shoemaker & E. Müll.	BEOFB 2301m	fresco	-	-	-	7.01 ± 0.05
38	*Thanatephorus cucumeris* (A.B. Frank) Donk	BEOFB 2401m	fresco	-	-	-	7.07 ± 0.04

“+“: screened activity detected

“-“: activity absent

Positive strains in pigment secretion assay were marked as color categories from ISCC-NBS color palette [30], indicating color of produced pigments; Color categories: s.YG–strong yellow green; s.O–strong orange; l.OY–light orange yellow; deep rO—deep reddish orange; m.YG–moderate yellow green; s.P–strong purple; m.OlG–moderate olive green; v.rO—vivid reddish orange; deep O–deep orange; l.OY–light orange yellow; brill.Y–brilliant yellow; brill.OY–brilliant orange yellow; v.R–vivid red

pH values of broth medium are represented as mean values of three measurements ± standard deviation

Conidial suspensions of the tested isolates were prepared by washing conidia from the surface of 7 day old malt extract agar (MEA) slants using sterile saline solution (0.9%,NaCl, HemofarmhospitaLogica) with 0.1% Tween 20 (Sinex Laboratory). Concentrations of conidia in the suspensions were calculated using the formula given in the manufacturer's guidelines (Reichert, Warner-Lambert Technologies):

$$\frac{number\;of\;conidia}{{mm}^{2}}\times 10000\times dilution$$

based on the number of conidia counted on a 1 mm^2^ surface of the hemocytometer used for this purpose (Reichert, Warner-Lambert Technologies). The suspensions were adjusted to a final concentration of approximately 1.0 × 10^5^ CFU ml^-1^ with sterile saline solution, and stored at −20°C. Prior to the experiments, conidial suspensions were cultured on solid MEA to check validity of the inocula and verify the absence of contamination.

### Biodegradative plate assays

All biodegradative plate assays were performed in triplicate.

#### Carbonate dissolution test

The potential ability of the tested microfungi to solubilize calcite was screened using CaCO_3_ glucose agar plates of the following composition, per litre of deionized water: calcium carbonate, 5 g; glucose, 10 g; agar, 15 g; and deionized water, 1000 ml. The prepared medium was sterilized at 121°C for 15 min, after which pH was adjusted to 8.0 with 10 N HCl and the mixture was cooled to 45°C with gentle stirring to resuspend CaCO_3_. After pouring into Petri plates, the plates were then kept in a cool place. Petri plates with agarized CaCO_3_ glucose agar were inoculated with 10 μl of fungal suspensions and incubated for 21 days at 25 ± 2°C (UE 500, Memmert). Positive strains displayed a clear zone around the colony, thus confirming calcite dissolution [27].

#### Acid and alkali production

To determine the capacity of fungi to affect the pH value of the substrate on which they grow, isolates were cultivated in a liquid minimal medium of the following composition, per litre of deionized water: sodium nitrate, 3 g; dipotassium phosphate, 1 g; magnesium sulphate heptahydrate, 0.5 g; potassium chloride, 0.5 g; iron (II) sulphate heptahydrate, 0.01 g; glucose, 10 g; and deionized water, 1000 ml [28]. Titration flasks with 100 ml of medium, sterilized at 114°C for 25 minutes and having its pH readjusted to 7.0 with 10 N HCl, were inoculated with 10 μl of fungal suspensions and incubated for three days on a platform shaker (Titramax 1000, Heidolph) under conditions of room temperature (22 ± 2°C) and rotation of 300 rpm. After the incubation period, cultures were filtered through Whatman No. 4 filter paper and a pH meter (pH CYBERSCAN 510, Eutech) was used to measure pH values of the filtrates, i.e., the culture medium. Measurements were performed in triplicate, with results presented as mean values of a number of repetitions with the standard deviation.

#### Casein hydrolysis assay

The proteolytic activity of fungal isolates was determined by cultivation on casein agar (CN) plates of the following composition, per litre of deionized water: sodium chloride, 5 g; peptone, 5 g; yeast extract, 3 g; agar, 15 g; and deionized water, 1000 ml. Skimmed milk (250 ml), fractionally sterilized daily (for 3 days) at 100°C for 30 min, was added to 750 ml of warmed sterilized medium (114°C for 25 min; pH readjusted to 6.8 with 4M NaOH), and homogeneously mixed: after cooling to approximately 45°C, the medium was then poured into Petri plates [9]. The centre of agarized medium was inoculated with 10 μl of fungal suspensions and the plates were incubated for 7 days at 25 ± 2°C. Following the incubation period, the agar plates were flooded with 5 ml of a 10% tannin solution to facilitate visualization of the casein hydrolysis zone [29].

#### Pigment secretion assay

The potential ability of the tested microfungal isolates to produce and secrete organic pigments under nutrient-limited conditions (ideally present on the surface of clean, properly maintained mural paintings), and consequently to induce alterations in the original colouration of the painted layer, was assayed by cultivation on Czapek-Dox minimal medium of the following composition, per litre of deionized water: sodium nitrate, 2 g; dipotassium phosphate, 1 g; magnesium sulphate heptahydrate, 0.5 g; potassium chloride, 0.5 g; iron (II) sulphate heptahydrate, 0.01 g; glucose, 10 g; agar, 20 g; and deionized water, 1000 ml [28]. The medium was sterilized for 25 minutes at 114°C, after which its pH was adjusted to 5.5 with 10N HCl. Petri plates inoculated with 10 μl of fungal suspensions, were incubated for 21 days at 25 ± 2°C. Secretion of fungal pigments was confirmed by changes in colour of the transparent medium. The colour of the produced pigment was determined by comparing it with the ISCC-NBS colour palette [30].

## Results

The deterioration potential of fungal isolates obtained from the sacral ambient is summarized in Table 1. Most of the isolates tested via biodegradative plate assays demonstrated at least one of the degradative abilities (71.05%), with *Penicillium bilaiae* testing positive in all four. The remaining 11 fungal isolates (28.95%) showed no deterioration capabilities and hence were considered not to partake in the complex process of fungal-induced deterioration of wall paintings via the tested mechanisms. These fungi likely induce deterioration through mechanical stresses created by hyphal penetration into the painted layers and mortar, or by means of other deterioration mechanisms, i.e., enzymatic degradation, biomineralization, etc.

**Fig 2:**
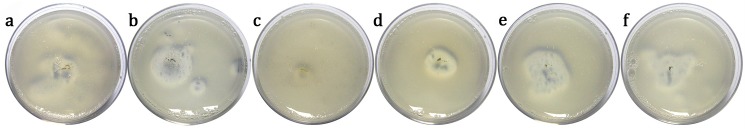
**Calcite dissolution induced by fungal growth and metabolism in cultures (day 21, CaCO_3_ glucose agar):** *Penicillium lanosum* BEOFB 1161m (a); *Aspergillus niger* (b); *A*. *europaeus* (c); *P*. *bilaiae* (d); *P*. *rubens* (e); *P*. *commune* (f).

Fungal-induced dissolution of calcium carbonate, i.e. the main component of mortar, the “building block” and carrier of painted layers in wall paintings, was detected in only seven out of 38 (18.42%) isolates, three of which were obtained from wall paintings (*Aspergillus europaeus*, *A*. *niger* and *Penicillium lanosum*) and four from the surrounding air (*P*. *bilaiae*, *P*. *commune*, *P*. *lanosum*, and *P*. *rubens*). Among the tested *Aspergillus* species, only 18.18% (2/11) had a transparent zone in the culture, while in the genus *Penicillium*, solubilization ability was observed for the majority of tested isolates (71.43%; 5/7) (Table 1). In all isolates that tested positive, a transparent solubilization zone could be detected as early as the third day of the incubation period, though fungal growth remained very restricted for a period of 21 days, with a small amount of produced mycelia and very restricted sporulation (Fig 2A–2F), most notably in the case of *A*. *europaeus*. Likewise, to judge from diameter of the observed CaCO_3_ dissolution zone, *A*. *europaeus* and *P*. *bilaiae* possess the lowest calcite solubilization potential.

**Fig 3:**
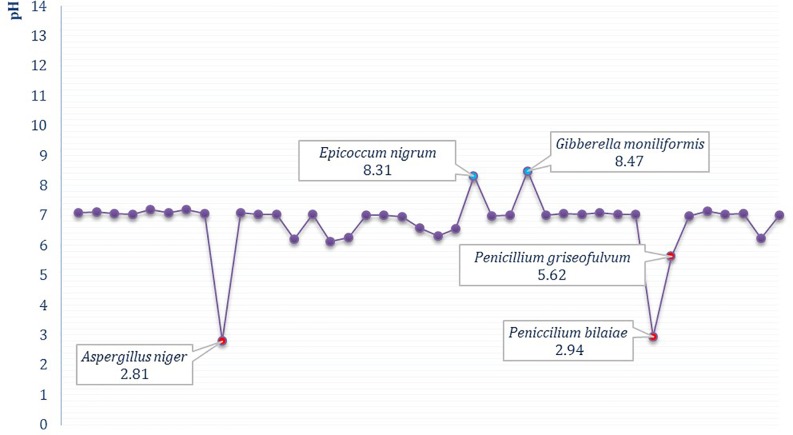
Production of acidic and alkaline metabolites in broth cultures of tested fungal isolates (day 3, Czapek-Dox minimal broth). Each dot on the chart corresponds to one of the screened isolates, with emphasis on fungi that considerably altered pH of the broth medium.

Short-term cultivation in a nutrient-limited broth medium showed that five out of 38 fungal isolates (13.16%) (Fig 3A–3E) were able to considerably alter pH of the broth medium, which ranged from 2.81 ± 0.47 to 8.47 ± 0.21 (Table 1). The lowest measured pH values were in cultures of *A*. *niger* (2.81 ± 0.47) and *P*. *bilaiae* (2.94 ± 0.33), followed by *P*. *griseofulvum*, with pH 5.62 ± 0.23. Growth and metabolism of only two species, *Gibberella moniliformis* and *Epicoccum nigrum*, caused an increase in alkalinity of the medium, with measured values in the pH range of 8.31 ± 0.17 to 8.47 ± 0.21. In the remaining 33 isolates (86.84%), only moderate changes of substrate pH values, in the range of one pH unit, were documented (Table 1).

**Fig 4:**
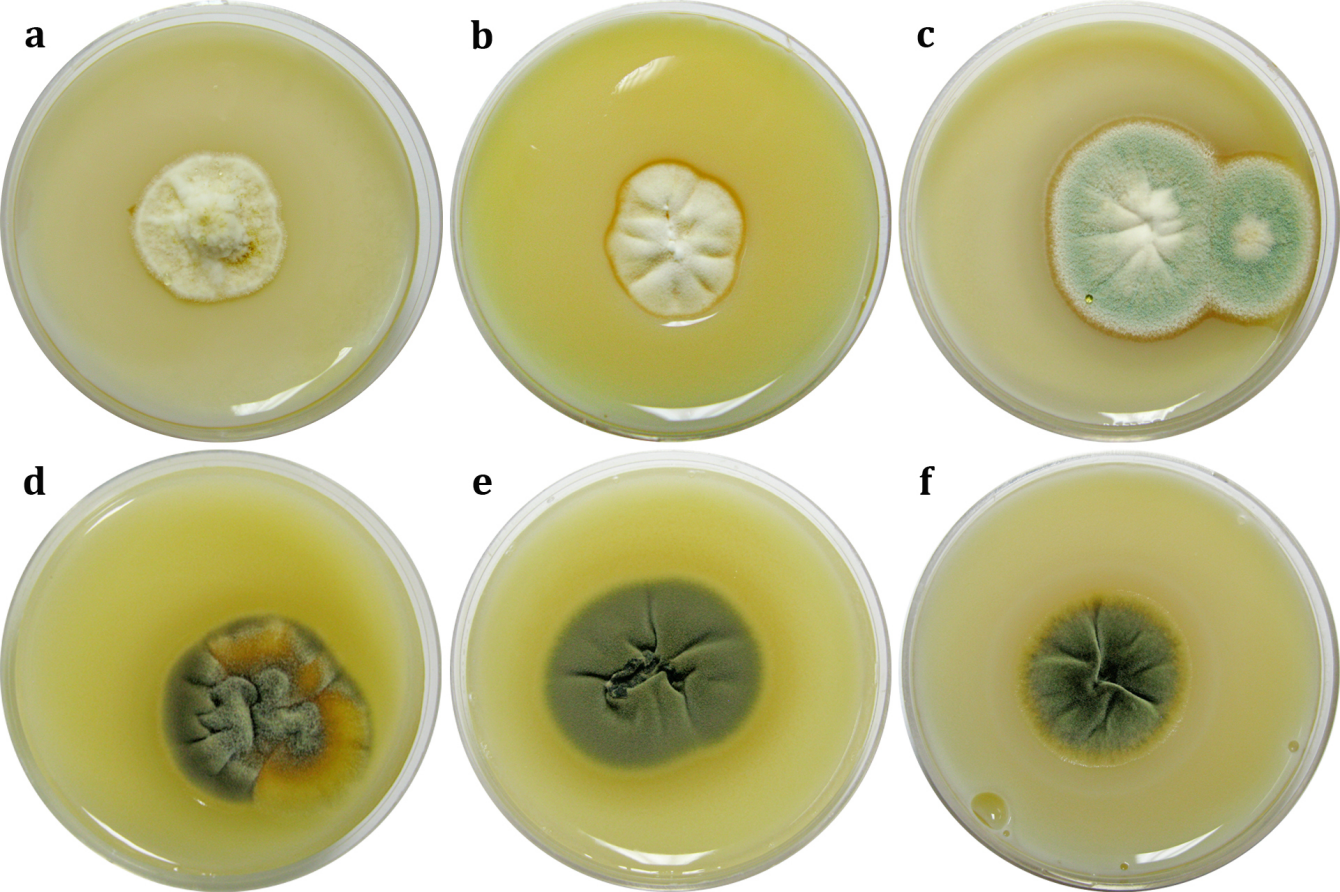
**Casein hydrolysis zones in cultures (day 7, casein nutrient agar):** *Aspergillus europaeus* (a); *A*. *versicolor* (b); *P*. *griseofulvum* (c); *Cladosporium cladosporioides* (d); *C*. *uredinicola* (e); *C*. *oxysporum* (f).

Out of the 38 tested isolates, a casein hydrolysis zone was observed in 16 (42.11%) (Table 1), mainly in the genus *Aspergillus* (63.64%; 7/11) (Fig 4A and 4B) and to a lesser extent in the genera *Penicillium* (28.57%; 2/7) (Fig 4C), *Cladosporium* (100%; 3/3) (Fig 4D–4F), and *Chaetomium* (66.67%; 2/3). Proteolytic activity was also detected in cultures of *Bjerkandera adusta* and *Phaeospheria avenaria*. The greatest diameters of the transparent zone were measured for *Cladosporium* species, with *C*. *cladosporioides* showing the largest zone, indicating the strongest proteolytic potential. Furthermore, in a culture of *C*. *cladosporioides* cultivated for one week on CN medium, morphological alteration in the guise of a partial change in colour of the colony, from dull green to shades of yellow and beige, was also observed (Fig 4D).

**Fig 5:**
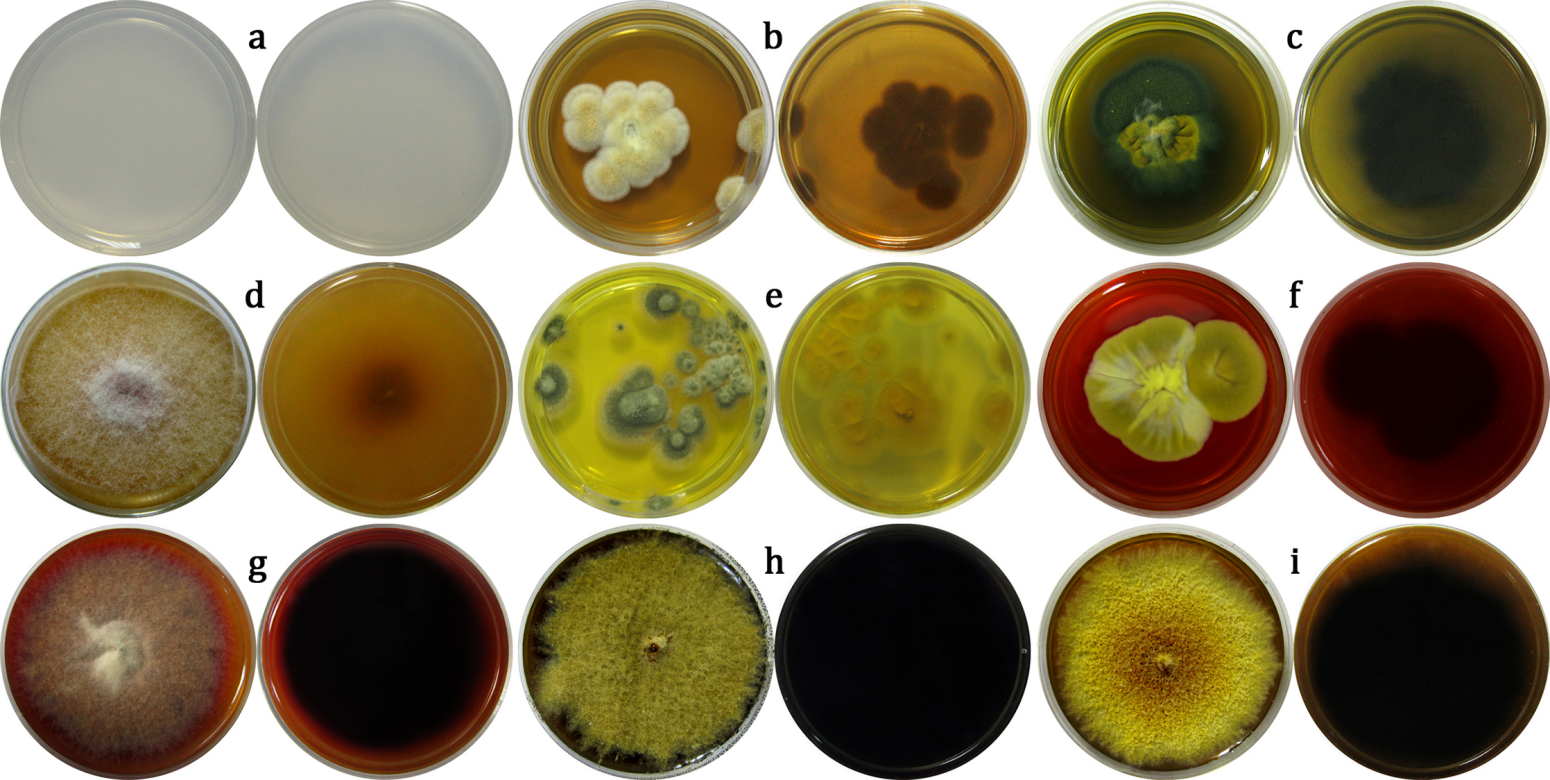
**Fungal pigment secretion in cultures (day 21, Czapek-Dox minimal medium):** Transparent non-inoculated plate (a); *Aspergillus flavipes* (b); *A*. *aureolatus* (c); *Gibberella moniliformis* (d); *Penicillium bilaiae* (e); *P*. *manginii* (f); *Epicoccum nigrum* (g, h, i).

Colouration of the transparent medium (Fig 5A) was observed in 15 isolates (39.47%) (Table 1). Most of them were of the genera *Aspergillus* (36.36%; 4/11) (Fig 5B and 5C) and *Penicillium* (57.14%; 4/7), while in isolates of other fungal genera, such as *Gibberella* (Fig 5D), pigment secretion was observed in only a few instances. Most of the isolates produced pigments in various shades of orange, while green and red pigments were present in three cultures. *Chaetomim murorum* and *P*. *bilaiae* (Fig 5E) were the only isolates with purple and bright yellow pigments. As many as 14 out of 15 isolates produced a sufficient amount of pigments to fully change colour of the transparent medium, although in cultures of the slow-growing fungi *A*. *creber*, *A*. *versicolor*, and *P*. *manginii* (Fig 5F), the process was very slow and full colouration could only be observed at the end of the incubation period. The greatest variety in shades and colours of produced pigments among different isolates of the same species was documented for *Epicoccum nigrum* (Fig 5G–5I). *Chaetomium murorum* was the only species with an isolate that produced small quantities of pigment, manifested as a purple halo around the colony margins. On the other hand, in the case of fungi from the genera *Alternaria* and *Cladosporium*, pigment production was present, but the produced melanin remained bound within the cell wall and was not secreted into the medium.

## Discussion

Limestone dissolution induced by fungal metabolites is a well-known and thoroughly studied natural phenomenon in the terrestrial environment [31], albeit less studied in regard to the possible damage it causes to carbonate substrata of cultural heritage monuments [32, 33]. Albertano and Urzì [27] stated that solubilization of limestone and consequent deposition of secondary carbonates induced by various microorganisms is the primary cause of structural alterations of carbonate substrata. However, though very important, only a few studies since the mid-nineties have complemented data on microbial diversity with appropriate biodegradative assays. In two very similar investigations, Pangallo et al. [9, 34] cultivated fungi isolated from stone monuments, indoor artwork, wall paintings and ambient air on CaCO3 glucose agar and showed that many species of the genera *Aspergillus* and *Penicillium* dissolve calcite, which fully corresponds with the results of our study. In both cases, only a small number of fungal isolates, 12.72 and 21.15%, respectively, mainly of these two genera, demonstrated a calcite solubilization ability. Apart from the work of Pangallo et al. [9, 34], experimental proof of calcite dissolution in CaCO_3_-enriched MEA inoculated with *A*. *niger* was provided by Sayer et al. [35]. On the other hand, *Doratomyces* sp. and *Paecilomyces* sp. isolated from variously coloured patinas of the Catacombs of St. Callistus and Priscilla in Rome (Italy) were the only fungi able to dissolve calcite when cultured on CaCO_3_ glucose agar, while all *Aspergillus* and *Penicillium* isolates tested negative [27]. Compared to all previous research, a recent work of Ortega-Morales et al. [36] showed the greatest percentage of fungi able to dissolve calcite, with more than 59% of fungi isolated from surface microhabitats of Mayan buildings testing positive when cultured on CMEA and CR2A-A selective media. As these two agarized nutrient media vary in composition with respect to the CaCO_3_ glucose agar used, fungal carbonate solubilization activity presumably depends not only on environmental factors and origin of the isolates, but on available sources of carbon and nitrogen as well. A similar conclusion was also reached in a study of fungal degradation of apatite, galena and obsidian minerals [37].This is likely due to the fact that the primary way carbonate dissolution occurs is through synthesis and secretion of various organic acids, although other mechanisms have also been proposed, for example enzymatic dissolution, ligand activity, and oxidation-reduction of redox-sensitive elements(only when CO_2_ is used as a carbon source for autotrophy, as CaCO_3_ is not a redox sensitive compound) [32, 38, 39, 40]. Albertano and Urzì [27] suggested that microfungi colonizing marble and limestone monuments use nutrients produced by phototrophic microorganisms to synthesize organic acids which dissolve CaCO_3_ from the substrate. According to Sterflinger [32] and Ortega-Morales et al. [36], only fungi that produce and secrete acids can dissolve CaCO_3_, and strong oxalic acid (C_2_H_2_O_4_) is the main “culprit” in most instances. Since organic acids are synthesized, as a by-product of oxidative metabolism during primary fungal metabolism, it is no wonder that in our study and many others as well [9, 34, 36], a transparent zone of CaCO_3_ dissolution was formed very early, i.e., during the first week of the incubation period. However, only in broth cultures of *A*. *niger* and *P*. *bilaiae* was acid production documented, via substantial changes in pH of the filtrate. It follows that in cultures of other positive isolates, CaCO_3_ dissolution either occurred as a result of acid production induced by the presence of Ca ions, or by one of the other mentioned mechanisms. In most cases, to produce acids, an abundant carbon source, i.e., sugars, is needed, since intensive growth results in production of more organic acids than is necessary for normal metabolism, the excess being excreted into the substrate. Cultured in Czapek-Dox minimal broth, an essentially oligotrophic medium, only a very small number of isolates, one of *Aspergillus* and two of *Penicillium*, caused considerable acidification of the broth medium, possibly owing to acid production. Many species of these two genera, isolated from artworks are known to synthesize acids, with *A*. *niger* being one of the strongest producers, primarily of oxalic, gluconic and citric acids [41, 42, 43]. *Penicillium bilaiae* is also a strong, producer of oxalic and citric acid, while *P*. *griseofulvum* is known to synthesize two acids: genistic and shikimic [44, 45]. And though results for these three fungi are in full accordance with previously published data, many of the tested species did not induce changes of substrate pH inspite of literature data acknowledging them as acid producers. Many of the micromycetes tested in our research have already been tested in other similar studies involving cultural heritage objects [28, 30, 41, 46, 47, 48, 49], where it was shown that numerous species from the genera *Aspergillus*, *Cladosporium*, and *Penicillium* genera are potent acid producers. However, some of these studies were performed utilizing nutrient media rich in glucose, so carbon source availability and abundance evidently constitutes a very important factor in acid metabolism. Moreover, several fungal isolates known to be acid producers in our experiment did in fact lower pH of the broth medium by values on the order, of one pH unit. However, on the basis of such modest changes in pH, it cannot be definitely concluded that acid synthesis occurred without additional analysis by HPLC.

On the other hand, in cultures of *Gibberella moniliformis* and *Epicoccum nigrum*, increase in the pH level of the medium was documented, possibly as a result of secretion of alkaline metabolites, such as NH_3_ and some polypeptides. Although species of the genus *Fusarium* are mainly known as producers of oxalic, fumaric, and succinic acid [41], results similar to those presented here, obtained with *Fusarium proliferatum* isolate AZhT01, from an ancient stone stela, and indicating an increase of pH when cultured in the same medium, were previously reported by Savković et al. [50]. It is important to note that while acids are generally referred to as "dangerous" metabolites for cultural heritage objects, it has been argued that deterioration can also incur via alkaline reactions, through degradation of nitrogen complexes and Na-salts of organic acids [51].

The casein hydrolysis assay applied in the present study (CN medium) was originally formulated as a simple and rapid method for screening proteolytic activity of microorganisms, so that adequate microbial strains, producers of important proteases, can be selected and further investigated for various uses in industrial biotechnology [29]. As the method entails use of a non-selective medium widely employed for isolation and cultivation of lactic acid bacteria, it recently also found application in other research areas, such as study of the biodegradative potential of the microbial community thriving on artwork [9]. Here it is presumed that the fungal extracellular enzyme casease, if produced, will degrade casein into polypeptides, peptides, and amino acids, making them available for absorption, which will result in loss of colour by the white milk protein and formation of a transparent zone around the colony. Our research indicates that many fungi, mainly species of the genera *Aspergillus* and *Cladosporium* (and *Chaetomium* and *Penicillium* species to a lesser extent), produce casease, which breaks the peptide bonds in casein, a finding that corresponds well with previously published data [9, 30, 52]. Where CN medium was applied in those studies, fungi from the genera *Aspergillus*, *Cladosporium*, and *Penicillium* were similarly characterized as the main producers of casease and as such the primary degraders of proteinaceous substrata of cultural heritage objects. And while the proteolytic activity of several fungi studied in these investigations (*A*. *flavus*, *A*. *niger*, *A*. *versicolor*, and *C*. *oxysporum*) is corroborated by our results in the study of Pangallo et al. [9] *C*. *cladosporioides* isolated from wall paintings and surrounding air of the Church of Saint Catherine in Velka Lomnica (High Tatra Mountains, Slovakia) lacked any observable transparent zone, which completely deviates from the findings obtained in our study, where it showed the highest proteolytic potential. Moreover, several *Aspergillus* isolates used in our study lacked a hydrolysis zone, although literature data [9, 30, 52] indicate them to be casease producers. Such discrepancies are possibly the result of varied cultivation conditions. Molitoris et al. [53] state that temperature and substrate salinity generally have very little impact on casease activity, while Rojas et al. [30] claim that discrepancies in detecting casease activity among various isolates of the same species are due to substrate pH, with fungi more likely to produce casease in substrates with lower pH. It should be noted that, some fungi, e.g., *A*. *niger* and *A*. *flavus*, are very tolerant to a wide range of pH and can thus produce enzymes whatever the acidity. Since casein agar medium has an approximately neutral pH value (6.8 ± 0.2), the absence of casein hydrolysis in known producers such as *A*. *oryzae* is not unexpected. As for *Chaetomium* isolates, the two tested species, *Ch*. *ancistrocladum* and *Ch*. *murorum*, both hydrolyzed casein, which is in accordance with the generally accepted view that this genus encompasses a large number of species that are strong producers of cellulolytic and proteolytic enzymes [54]. However, another screened strain of *Ch*. *murorum* did not produce casease, this may be the result of changes in morpho-physiological features of the isolate induced by growth on frescoes, which are an extreme type of environment. It is also worth noting that, according to Vermelho et al. [55], diameter of the hydrolysis zone of CN medium indicates the amount of produced casease. While this type of assessment cannot be considered exact without direct measurement of the quantity of produced casease, many authors [29] consider the given method suitable for obtaining general insight into the proteolytic capacity of tested fungal isolates. With that in mind, attention should be paid to the fact that the screened *Cladosporium* isolates had the largest diameters of the hydrolysis zone, which suggests that they possess the highest proteolytic activity and points to them as the primary degraders of protein components within mortar and painted layers of wall paintings. The present study particularly emphasizes the importance of cooperation between mycologists and conservators in the exchange of knowledge regarding fungal proteolytic capacities and protein binders commonly used in secco painting in a given region, since their use even today is largely the result of an insufficient level of communication. Only in this way, by working together in selecting appropriate binders for restoring damaged wall paintings, can re-colonization of restored surfaces be prevented and long-lasting and effective safeguarding of murals be ensured.

Micromycetes are known producers of a large variety of organic pigments, each with its own structure, composition, and colour, which are regulated by a number of things such as the available carbon and nitrogen source, metals in the substrate, light (UV), and other environmental factors that limit growth [19]. Many isolates have been shown to secrete variously coloured pigments, as indicated by changes in colour of the employed transparent medium, something which has been previously confirmed several times under nutrient-limited conditions with many *Aspergillus*, *Chaetomium*, *Cladosporium*, *Fusarium*, and *Penicillium* species isolated from wall paintings, documents, books, paintings, and photographs [11, 28, 30]. Furthermore, it was recently shown that same fungal species, albeit isolated from a variety of substrata, can produce pigments of different colour or lack pigment production at all [30], which would account for the inconsistencies in colour production documented here and in previously published research. An interesting finding is the purple pigment detected in a culture of *Ch*. *murorum*. Which pigment this is cannot be said without chemical analysis, although it is known that some species of the genus *Chaetomium*, viz. *Ch*. *cochliodes* and *Ch*. *globosum*, when cultured on various nutrient media produce a very small amount of the purple pigment cochliodinol (C_32_H_32_N_2_O_4_) proven to possess strong antimicrobial activity [56].

An additional complication is the fact that produced pigments can be secreted into the substrate or (more commonly) bound within the protoplasm or incorporated into composition of the cell wall of hyphae and spores, which must be ascertained via light microscopy before adequate removal methods can be applied [19]. Coloured dry conidia present in mass quantities can be easily removed by simple vacuum treatments; however, to remove stains formed by secretion of pigment into the substrate, solubility of the given pigment must be known. This is usually not the case, as the chemical nature of the pigment is rarely determined in conservation practice. However, pigment secretion assay in combination with chemical analysis could provide essential information on the structure and solubility of the most commonly secreted pigments, which would go a long way toward selecting an appropriate solvent and eliminate the need for totally unacceptable and aggressive methods such as use of 1N KOH, 5% NaOCl, 30% H_2_O_2_, H_2_O_2_/Cu^2+^ mixture, UVA radiation, etc. [19].

Based on all of the above, it can be concluded that the vast majority of filamentous fungi have a very important role in the decay of wall painting components via carbonate dissolution, proteolysis, and acid and alkaline action, usually manifested in cracking, peeling, and loss of microfragments, as well as in impairment of aesthetic appearance by secretion of coloured metabolites. It should be noted, however, that some common constituents of the mycobiota of frescoes are nothing more than harmless allochthonous transients. For these reasons, detection of fungal growth on painted layers or the presence in the surrounding air of propagules belonging to species with a now known biodegradative potential should be treated with particular care and appropriate remedial measures in the form of removal of the detected growth and application of microclimate control. Furthermore, introduction of fast and simple biodegradative plate assays as an integral part of the protocol of a modern system for the protection and conservation of artworks should be mandatory, since insight into the total spectrum of physiological features of fungi, common inhabitants of sacral ambients, is a prerequisite for precise assessment of the amount of risk they pose to mural paintings.

## Conclusions

The obtained results clearly demonstrate that the ambient air and deteriorated wall paintings of the old Church of the Holy Ascension are contaminated with several fungal strains associated with various biodeteriorative effects that most likely are partially or completely the cause of the documented damage. The present study also emphasizes the importance of knowledge about the biodegradative characteristics of fungal dwellers present on or in the immediate vicinity of artworks, as it represents the basis for precise risk assessment and formulation of a set of recommendations to cultural institutions regarding the implementation of preventive and remedial treatments. When known deteriogenic fungi are detected, measures in the guise of monitoring and frequent cleaning, introduction of adequate ventilation, and indoor climate management, must be carried out in order to prevent their establishment and subsequent deterioration of valuable artworks.
